# The root pathogen *Aphanomyces euteiches* secretes modular proteases in pea apoplast during host infection

**DOI:** 10.3389/fpls.2023.1140101

**Published:** 2023-03-27

**Authors:** Andrei Kiselev, Laurent Camborde, Laura Ossorio Carballo, Farnusch Kaschani, Markus Kaiser, Renier A. L. van der Hoorn, Elodie Gaulin

**Affiliations:** ^1^ Laboratoire de Recherche en Sciences Végétales (LRSV), Université de Toulouse, CNRS, UPS, Toulouse INP, Auzeville-Tolosane, France; ^2^ The Plant Chemetics Laboratory, Department of Plant Sciences, University of Oxford, Oxford, United Kingdom; ^3^ ZMB Chemical Biology, Faculty of Biology, University of Duisburg-Essen, Essen, Germany

**Keywords:** *Aphanomyces*, root rot, plant pathogen, proteases, apoplast, extracellular, activity-based proteomics

## Abstract

To successfully colonize the host, phytopathogens have developed a large repertoire of components to both combat the host plant defense mechanisms and to survive in adverse environmental conditions. Microbial proteases are predicted to be crucial components of these systems. In the present work, we aimed to identify active secreted proteases from the oomycete *Aphanomyces euteiches*, which causes root rot diseases on legumes. Genome mining and expression analysis highlighted an overrepresentation of microbial tandemly repeated proteases, which are upregulated during host infection. Activity Based Protein Profiling and mass spectrometry (ABPP-MS) on apoplastic fluids isolated from pea roots infected by the pathogen led to the identification of 35 active extracellular microbial proteases, which represents around 30% of the genes expressed encoding serine and cysteine proteases during infection. Notably, eight of the detected active secreted proteases carry an additional C-terminal domain. This study reveals novel active modular extracellular eukaryotic proteases as potential pathogenicity factors in *Aphanomyces* genus.

## Introduction

Root rot diseases are a major global threat to the productivity of agricultural crops. The term ‘root rot’ has been widely used to describe a group of diseases characterized by softening and necrosis of the roots, producing a broad spectrum of lesions of various colors and sizes (Sharma et al., 2022). The widely spread oomycetes and fungi are the most prevalent soil-borne root rot pathogens (Dean et al., 2012; Becking et al., 2021). *Aphanomyces euteiches* root rot (ARR) disease is one of the major limiting factors in the cultivation of North American (Papavizas and Ayers, 1974; Wu et al., 2018) and European pea (Quillévéré-Hamard et al., 2018; Quillévéré-Hamard et al., 2020), with some occurrence of this disease in Australian faba bean cultures (Watson et al., 2013).

Filamentous plant pathogenic oomycetes secrete several types of pathogenicity factors to facilitate infection, such as small secreted proteins (SSP), cellulose-binding proteins (CBELs) or plant DNA-damaging proteins (Gaulin et al., 2006; Ramirez-Garcés et al., 2016; Camborde et al., 2022). Before entering the root cells, these pathogens, including *A. euteiches*, may pass the apoplast. Due to its extracellular nature, the apoplast is involved in the perception and transduction of environmental signals (for a review, see Farvardin et al., 2020). On plant microbe detection, the plant cell wall is modified and the fluidic apoplast becomes a harsh environment equipped with antimicrobial compounds and various types of enzymes to restrict pathogen infection (Jashni et al., 2015; Dora et al., 2022). Basically, to survive in plant apoplast, phytopathogens depend on their ability to harvest nutrients, to hide from the host surveillance system and to attenuate host defense responses. Therefore, these pathogens produce cell-wall degrading enzymes (CWDE) and evolved molecular mechanisms to permit hiding, inhibition of defense-induced components, and detoxification/degradation of host components (Rocafort et al., 2020). Secreted proteases perform the two-last categories of actions and are present in the extracellular space of infected plant tissues. These proteases are found to originate from both the host and the pathogen (Jashni et al., 2015; Paulus et al., 2020).

Plant-secreted proteases are suspected to play major role during oomycete infection and numerous plant proteases are highly upregulated during infection, exemplified by aspartic protease StAsp from potato (Guevara et al., 2005) or the P69B serine protease from tomato (Tian et al., 2004; Paulus et al., 2020). Recent evidence has shown that proteases from oomycetes might also have a role to play during plant invasion. Expression profile of *in-silico* identified metalloprotease from *Phytophthora infestans* pinpointed a dozen of enzymes that potentially affect virulence of the pathogen (Schoina et al., 2021). Knockout-mutants of two secreted cysteine proteases from *Phytophthora parasitica* (PpCys44, 45) present a reduced virulence during *Nicotiana benthamiana* infection, while overexpression of both proteases in the plant apoplast triggers cell death (Zhang et al., 2020). In addition, a general counter-defense strategy used by invading oomycetes relies on the inhibition of host proteases. *P. infestans* secretes a large group of cystatin-like inhibitors (EPICs) known to target tomato Pip1, Rcr3 and C14 (Tian et al., 2007; Song et al., 2009; Kaschani et al., 2010; Van der Hoorn, 2011). Likewise, the Kazal-like serine protease inhibitors EP1 and EPI10 from *P. infestans* target tomato P69B subtilase (Tian et al., 2004, 2005). Interestingly, the *A. euteiches* genome, in addition to having numerous CWDE, is characterized by a large representation of putative extracellular proteases (Gaulin et al., 2018; Kiselev et al., 2022), suggesting a key role in infection.

To characterize the oomycete enzymes contributing to virulence, Meijer et al. (2014) performed a proteome profiling of the secretome from *P. infestans* grown on a plant-based medium and identified one aspartic protease, four cysteine proteases and two metalloproteases (Meijer et al., 2014). Similar studies on other *Phytophthora* species did not identify any proteases in the secretome (Severino et al., 2014; McGowan et al., 2020). These apparently contradicting results suggest that untargeted *in vitro* methods may be not ideally suited for protease identification. Activity-based protein profiling and mass spectrometry (ABPP-MS) uses highly selective active-site targeted chemical probes to label and characterize *in vivo*, active proteins including proteases (van der Hoorn et al., 2004; Kaschani et al., 2009). A set of probes were developed to target the different families of proteases, including papain-like cysteine proteases (PLCP), serine proteases, proteasomes, and metalloproteases (van der Hoorn, 2011; Morimoto and van der Hoorn, 2016). ABPP-MS has been used to identify a proteolytic cascade that activates immune proteases in tomato (Paulus et al., 2020), and to characterize secreted inhibitors from various pathogens (Shabab et al., 2008; Kaschani et al., 2010; Shindo et al., 2016).

Here we assessed whether the large repertoire of predicted *A. euteiches* proteases are active during host infection using ABPP-MS on infected-pea roots. We firstly defined the repertoire and genome organization of *A. euteiches* secreted proteases and evaluated their expression upon host infection. Then we identified 35 microbial proteases, among these, eight were original composite secreted proteases with a proteolytic domain associated to a non-catalytic domain that shows binding properties either for lipids or for carbohydrates. This work demonstrates ABPP-MS as an efficient *in vivo* tool to quickly substantiate genomics prediction of microbial pathogenicity factors. It allows the identification of original oomycete modular extracellular proteases, secreted by *A. euteiches* in the apoplastic host space in order to initiate the disease process.

## Materials and methods

### *A. euteiches* genome mining

Genome assembly (SRA accession SPR355760), predicted proteome sequence, and expression data (RNASeq) of *A. euteiches* ATCC201684 were accessed through the AphanoDB database (https://www.polebio.lrsv.ups-tlse.fr/aphanoDB/). The peptidases of *A. euteiches* were extracted as proteins harboring GO:0008233 and its child terms. To classify the genes into multigene families, the Markov Clustering Algorithm was applied (inflation rate 1,5) to cluster pairwise blast results (e-value < 1e^−30^). Tandemly repeated genes were identified as adjacent genes (blast e-value <1e^−80^, coverage > 80%). Microsynteny search was performed using OGOB browser (McGowan et al., 2019) and FungiDB (Basenko et al., 2018) resource using the best blast hit of the corresponding organism. Secreted proteases were identified as those with a predicted signal peptide using SignalP v.5 (Almagro Armenteros et al., 2019) and those without transmembrane helices were predicted using TMHMM v.2.0 (Krogh et al., 2001).

For C1A cysteine-proteases phylogeny tree reconstruction, *Phytophthora infestans* T30-4 and *Saprolegnia parasitica* CBS223-65 sequences were downloaded from the FungiDB repository (Basenko et al., 2018). The proteins of the different genomes were assigned with PFAM domain (PF00112) using InterProScan search (Sperschneider et al., 2015). The PF00112 domains were extracted and the phylogenetic tree was constructed and visualized in CLC Main Workbench v7.8.1(Qiagen), using ClustalW alignment and Neighbor-Joining (NJ) method with default parameters and bootstrap value of 1000.

### Whole expression analysis (RNASeq)

Previously generated RNASeq reads of *M. truncatula* A17-Jemalong infected with *A. euteiches* ATCC201684 at 1, 3, 9 days post infection and *A. euteiches* mycelium (Gaulin et al., 2018) are accessible at NCBI SRA under reference SPR355760. The raw data were trimmed with TrimGalore (v.0.6.5) (https://github.com/FelixKrueger/TrimGalore) with cutadapt and FastQC options, and mapped to *M. truncatula* cv Jemalong A17 reference genome v.5.0 using Hisat2 (v.2.1.0) (Kim et al., 2019). Samtools (v.1.9) algorithms ‘fixmate’ and ‘markdup’ (Li et al., 2009) were used to clean alignments from duplicated sequences. Reads were counted with HTseq (v.0.9.1) (Anders et al., 2015) using the reference GFF file (Kiselev et al., 2022). The count files were normalized and differentially expressed genes (DEGs) were identified using the DESeq2 algorithm (Love et al., 2014).

### Plant material, microbial strains and growth conditions

All experiments were carried out on the Précovil variety of *Pisum sativum* produced by the company Vilmorin (St Quentin Fallavier, France). Before germination, seeds were sterilised for 30 s in 96% EtOH, and 5 min in 5% bleach solution. After germination, the seeds were planted in 300 ml sterile pots filled with zeolite (1–5 mm fraction) and Fåhraeus media (Fåhraeus, 1957), supplemented with 5 mM NH_4_NO_3_ as nitrogen supplement. Zoospores of *A. euteiches* ATCC201684 were prepared as described elsewhere (Ramirez-Garcés et al., 2016). The plantelets were infected with 10^5^ zoospores per plant one day after transfer to zeolite pots. Roots were harvested 15 days after infection. Pea apoplastic fluid was extracted using 3-times vacuum infiltration with ice-cold water. Two bars of pressure were applied 3 times during 10 min. Infiltrated roots were dried by rolling in a paper towel, placed in a 20 ml syringe and then introduced into a 50 ml falcon tube followed by centrifugation at 4°C, 2000 rpm with a slow acceleration/deceleration program.

### Microscopy

Primary and upper secondary roots of pea infected or not by *A. euteiches*, were collected at 15 dpi for microscopic analysis. The primary root was placed directly on a holder and the secondary roots were embedded in 2,5% agarose and sliced using a vibrating-blade microtome (Leica VT1000 S) to 100 μm thickness. To specifically stain *A. euteiches* hyphae, wheat germ agglutinin coupled to Alexa Fluor 488 conjugate (WGA-488, Invitrogen) was used. Briefly, the specimens were stained in a 10 μg/ml staining solution for 5 min at room temperature and directly placed in a water drop on a microscope slide and observed using a confocal microscope. A confocal laser scanning microscope (Leica TCS SP8 operated on the LAS X software platform) was used to image the samples. Alexa Fluor 488 was detected between 500-565 nm using an OPSL 488 nm laser. Specimens were observed using a 10X dry objective (HC PL FLUOTAR 10x/0.30). All images were processed using ImageJ software version 1.53.

### Labelling active apoplastic hydrolases and affinity purification

Three ml aliquots of pea apoplastic fluid (AF) per treatment were labelled with 4 μM of FP-biotin (Sigma) and 4 μM DCG04 (MedKoo Biosciences) during 4 h at room temperature under slow rotation (10 rpm). The reaction was buffered using 50 mM NaAc (pH 5) and 5 mM of dithiothreitol (DTT). No-probe control (NCP) samples were identical to labelled samples but instead of probes, an equal volume of DMSO was added to 3 ml of AF. Labelling reactions were stopped by chloroform/methanol precipitation. Cold chloroform+water+methanol mixture in the volume ratio 1:3:4 was added to the samples and mixed thoroughly. The precipitated samples were stored in the freezer at -20°C. Next, the samples were centrifuged at 3000 g for 30 min at 4°C. The aqueous top phase was removed without disturbing the interphase in which the proteins were present. Four volumes of cold methanol were then added, and the samples were centrifuged again for 45 min at 3000 g at 4°C. The supernatant was removed without disturbing the pellet and the precipitated proteins were left to dry at room temperature. The precipitated proteins were resuspended with 2 ml of 1.2% sodium dodecyl sulphate (SDS) phosphate saline buffer (PBS) (Life Technologies, 18912-014) for at least 40 min. The samples were then sonicated in a sonication bath at maximum power for 10 min. A further 5 ml of PBS was added to the samples and the proteins were denatured in a water bath at 90°C for 5 min. A further 3 ml of PBS was added to the samples to lower the SDS concentration to below 0.2%. To enrich the labelled proteins, 130 μl of PBS-washed avidin beads (Sigma, A9207) were added to each sample, including the NPC. The beads were incubated with resuspended proteins for 1 h at room temperature under rotation. The beads were then centrifuged for 1 min at 400 g and the supernatant was discarded. The Avidin beads were then washed 5 times with 10 ml of 1% SDS PBS buffer to remove nonspecific protein-beads interactions, and 3 times with 10 ml of ultra-pure HPLC-MS grade water. Finally, the beads were transferred into 2 ml LoBind protein tubes (Eppendorf, Z666505-100EA).

### On-bead digestion and peptide purification

250 μl of 8 M Urea in 50 mM TrisHCl (pH 8) was added to the beads. The proteins were reduced by adding 500 mM of TCEP and incubating samples at 56°C for 30 min while shaking. The samples were then cooled to room temperature before the alkylation step. 30 μl of 500 mM chloroacetamide was added and alkylation was performed at 36°C for 30 min in the dark. The samples were centrifuged at 2000 rpm for 3 min and the supernatant was removed. A vial of 20 μg of LysC endopeptidase enzyme (Wako, 125-02543) was resuspended into 1220 μl of 1 M urea in 50 mM Tris-HCl pH 8. 80 μl of this resuspended LysC was added to each sample. The tubes were sealed with a parafilm and LysC digestion was performed overnight at 37°C while shaking. The following day, trypsin endopeptidase (Trypsin gold MS grade Promega V5280) was reconstituted following manufacturer’s instructions in 50 mM NaAc pH 5. 20 μg of the reconstituted trypsin was added to 1200 μl of 50 mM Tris-HCl pH 8. 80 μl of the diluted trypsin was added to each sample (2 μg per sample) and incubated for at least 8 h at 36°C. After trypsin digestion, tryptic peptides present in the supernatant were recovered in a new Lobind protein tube. Prior to mass spectrometry analysis of the peptidic composition, the tryptic peptides were purified using 100 μl Agilent Bond Elut OMIX pipette tips for micro extractions (Agilent, A57003100) using a 1 ml syringe coupled with a 1000 μl cut filter tip to push buffers and samples through the C18 column.

### LC-MS/MS

Experiments were performed on an Orbitrap Fusion Lumos instrument (Thermo) coupled to an EASY-nLC 1200 liquid chromatography (LC) system (Thermo). LC was operated in the one-column mode. The analytical column was a fused silica capillary (75 µm × 46 cm) with an integrated PicoFrit emitter (New Objective) packed in-house with Reprosil-Pur 120 C18-AQ 1.9 µm resin (Dr. Maisch). The analytical column was encased by a column oven (Sonation) and attached to a nanospray flex ion source (Thermo). The column oven temperature was adjusted to 50 °C during data acquisition. The LC was equipped with two mobile phases: solvent A (0.1% formic acid, FA, in water) and solvent B (0.1% FA, 20% water and 80% acetonitrile, ACN). All solvents were of UPLC grade (Honeywell). Peptides were directly loaded onto the analytical column with a maximum flow rate that would not exceed the set pressure limit of 980 bar, usually around 0.6–0.8 µL/min. Peptides were subsequently separated on the analytical column by running a 140 min gradient of solvent A and solvent B (start with 8% B; gradient 8% to 35% B for 95 min; gradient 35% to 44% B for 20 min; gradient 44% to 100% B for 10 min and 100% B for 15 min) at a flow rate of 250 nl/min. The mass spectrometer was operated using Orbitrap Fusion Lumos Tune Application (version v3.3.2782.28) and Xcalibur (v4.3.73.11). The mass spectrometer was set in the positive ion mode. Precursor ion scanning was performed in the Orbitrap analyzer (FTMS; Fourier Transform Mass Spectrometry) in the scan range of m/z 375–1750 and at a resolution of 120000 with the internal lock mass option turned on (lock mass was 445.120025 m/z, polysiloxane) (Olsen et al., 2005). Product ion spectra were recorded in a data dependent fashion in the ion trap (ITMS) in variable scan range and at a rapid scan rate. The ionization potential (spray voltage) was set to 2.3 kV and the ion transfer tube temperature was 275°C. Peptides were analyzed using a repeating cycle (cycle time = 3 s) consisting of a full precursor ion scan (4.0 × 10^5^ ions or 50 ms) and a variable number of product ion scans (1.0 × 10^4^ ions, injection time set to ‘auto’); peptides were isolated based on their intensity in the full survey scan (threshold of 5000 counts) for tandem mass spectrum (MS2) generation that permits peptide sequencing and identification. Stepped Higher-energy collisional dissociation (HCD) energy was set to 20, 35 and 40% for the generation of MS2 spectra. During MS2 data acquisition, the dynamic ion exclusion was set to 25 s (mass tolerance ±10 ppm) and a repeat count of 1. Ion injection time prediction, preview mode for the FTMS, monoisotopic precursor selection and charge state screening (charge states: 2 –6) were enabled.

### Peptide and protein identification using MaxQuant

RAW spectra were submitted to Andromeda (Cox et al., 2011) search in MaxQuant (2.0.2.0) using default settings (Cox and Mann, 2008). Label-free quantification and match-between-runs was activated (Cox et al., 2014). The MS/MS spectral data were searched against the *A. euteiches* database (Kiselev et al., 2022) and the *P. sativum* database (Kreplak et al., 2019). All searches included a contaminants database search (as implemented in MaxQuant, 245 entries). The contaminants database containing known MS contaminants was included to estimate the level of contamination. Andromeda searches allowed oxidation of methionine residues (16 Da) and acetylation of the protein N-terminus (42 Da) as dynamic modifications and the static modification of cysteine (57 Da, alkylation with iodoacetamide). Enzyme specificity was set to ‘Trypsin/P’ with two missed cleavages allowed. The instrument type in Andromeda searches was set to Orbitrap and the precursor mass tolerance was set to ±20 ppm (first search) and ±4.5 ppm (main search). The MS/MS match tolerance was set to ±0.5 Da. The peptide spectrum match FDR and the protein FDR were set to 0.01 (based on target-decoy approach). The minimum peptide length was 7 amino acids. For protein quantification, unique and razor peptides were allowed. Modified peptides were allowed for quantification. The minimum score for modified peptides was 40. Label-free protein quantification was switched on, and unique and razor peptides were considered for quantification with a minimum ratio count of 2. Retention times were recalibrated based on the built-in nonlinear time-rescaling algorithm. Within parameter groups, MS/MS identifications were transferred between LC-MS/MS runs with the ‘match between runs’ (MBR) option in which the maximal match time window was set to 0.7 min and the alignment time window set to 20 min. The quantification was based on the ‘value at maximum’ of the extracted ion current. At least two quantitation events were required for a quantifiable protein. Further analysis and filtering of the results was done in Perseus v1.6.10.0. (Tyanova et al., 2016). For quantification, we combined related biological replicates to categorical groups and investigated only those proteins that were found in at least one categorical group in a minimum of 3 out of 4 biological replicates. Comparison of protein group quantities (relative quantification) between different MS runs is based solely on the LFQ’s as calculated by MaxQuant, MaxLFQ algorithm (Cox et al., 2014).

## Results

### *A. euteiches* encodes numerous secreted proteases, tandemly duplicated in the genome

**Figure 1:**
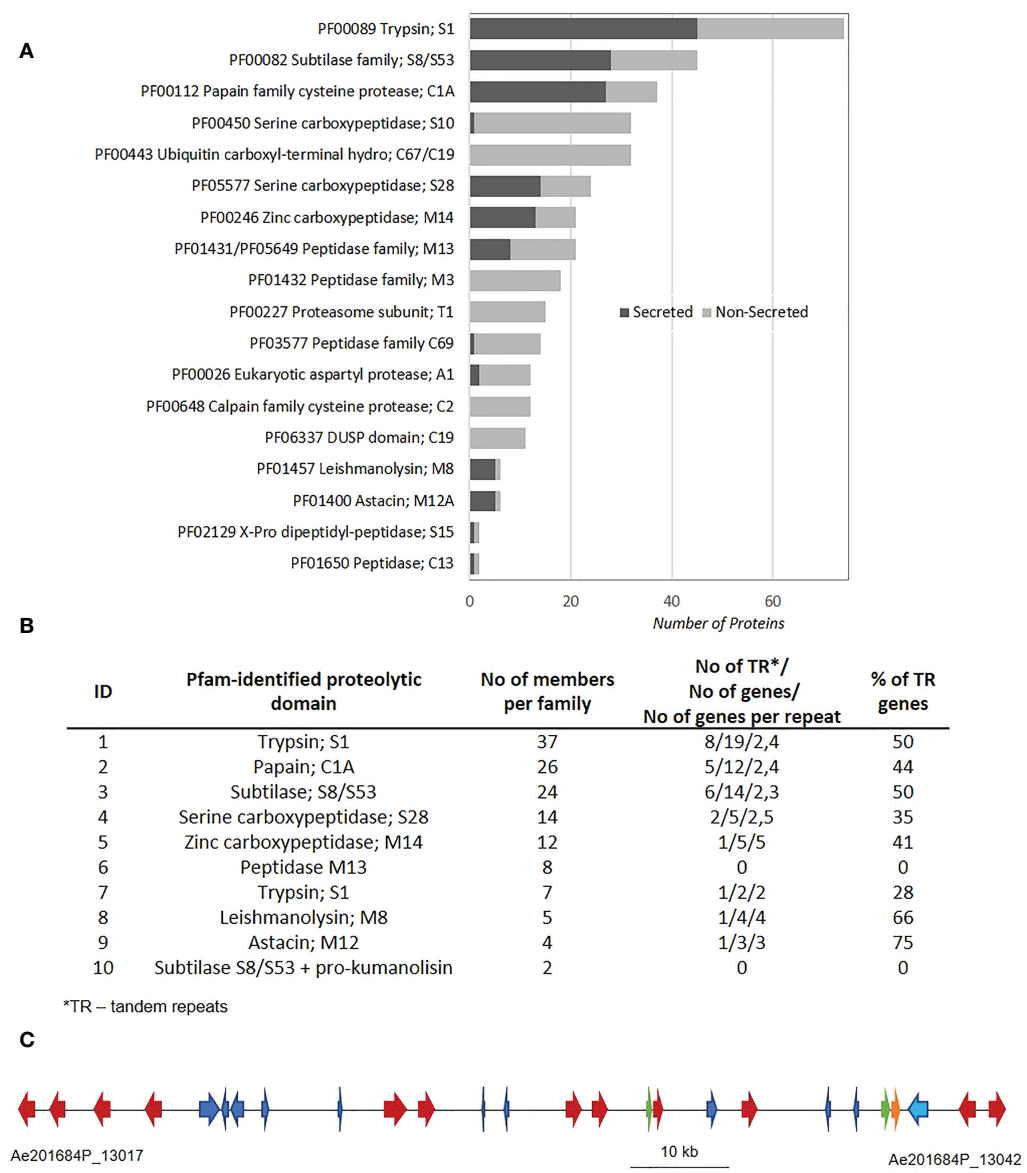
Mining of protease sequences from *A. euteiches* genome. **(A)** Repartition of proteolytic enzymes between secreted and non-secreted proteins in *A. euteiches* ATCC201684 genome. Proteolytic domains determined by InterProScan software against Pfam database. Secreted proteins (dark-grey) correspond to proteins with a predicted signal peptide (+SP) and without a predicted transmembrane domain (-TM). Data extracted from AphanoDB database (Madoui et al., 2007; Gaulin et al., 2018). **(B)** Distribution of secreted proteolytic enzymes in multigene families and tandem repeats in *A. euteiches*. Multigene families determined using Markov Cluster Algorithm (MCL) with a blast e-value < 1e^−30^, MCL inflation rate 1.5. Proteins considered in Tandem Repeats (TR), when having adjacent copy (blast e-value < 1e^−80^, coverage > 80%). **(C)** A 97 kb genome region (between Ae201684P_13017 and Ae201684P_13042 genes) in 2,7 Mb contig of *A. euteiches* enriched in tandemly repeated secreted subtilases (multigene family 3). The cluster contains 26 genes, corresponding to 11 secreted subtilases (red arrows), 1 non-secreted subtilase (orange arrow), 2 small secreted proteins (SSP, green arrows) and 13 non-secreted proteins with various functions (blue arrows). See **Supplementary Table S4** for the detailed description of proteins present in the cluster.

The AphanoDB, a database dedicated to the genus *Aphanomyces* (https://www.polebio.lrsv.ups-tlse.fr/aphanoDB/), contains 518 proteins with a PFAM-based GO Peptidase activity (GO:0008233), with trypsin S1 being the largest family (74 genes) in *A. euteiches* ATCC201684 strain (**Supplementary Table S1**). In a previous global analysis of the secretome we reported a large number of secreted proteases compared to the phytopathogen oomycete *P. infestans* (Gaulin et al., 2018; Kiselev et al., 2022). Accordingly, in this work we identified 151 proteins with a predicted signal peptide (+SP) and no transmembrane domain (-TM). Secreted proteases account for 28,5% of the total set of proteases indicating their significant enrichment in the secretome (Fischer exact’s test p < 0,05), and represent 10% of a total *A. euteiches* secretome. As illustrated in **Figure 1A**, more than 80% of secreted proteases correspond to five families based on PFAM domains: serine proteases from S1, S8/S53, S28 (trypsin, subtilase and carboxypeptidase), papain-like cysteine proteases C1A (PLCPs) and metalloproteases M14. In all these five families, the number of secreted proteases is greater than non-secreted, as in metalloproteases M8 and M12A families. The *A. euteiches* secretome has very few carboxypeptidase (S10) X-Pro dipeptidyl-peptidase (S15), cysteine peptidases (C69 and C13) and aspartyl proteases (A1) and no M3 peptidases (**Supplementary Table S2**). Thus, the secretory repertoire of proteases in *A. euteiches* spans thirteen families among the 281 described in MEROPS.

Since tandem duplication of genes is a driving force for the expansion of oomycete sequences related to pathogenicity (McGowan et al., 2019), we looked for the genomic organization of the secreted proteases within the *A. euteiches* genome. Markov cluster algorithm (MCL) grouped the 138 secreted proteases (92%) from the genome sequence of *A. euteiches* into 10 multigene families (blast e-value < 1e^−30^, MCL inflation rate of 1,5), with sizes ranging from 2 members to 37 per family (average of 14) (**Figure 1B**). Only twelve secreted proteases did not present any paralog and were considered as singletons (**Supplementary Table S3**). We identified tandemly repeated proteases within each family by looking for proteins with an adjacent copy (blast e-value < 1e^−80^, coverage > 80%). For eight out of ten families, a large proportion (over 28%) of the genes were found to be tandemly replicated, while the two small multigene families containing metallopeptidases M13 and subtilases, and pro-kumanolisin prodomain, did not contain any tandem duplications. The tandem duplication rate of secreted proteases is in the range 33–60% in *A. euteiches*, while an average rate of 4–14% is reported for the whole genome in oomycetes (McGowan et al., 2019).

The identification of multigene families with a high proportion of tandemly repeated genes prompted us to localize the family members in the genome of *A. euteiches.* For each multigene family, a genome region consists mainly of the family members. As illustrated in **Figure 1C**, within a 97 kb genome region consisting of 26 genes, twelve correspond to secreted subtilases from the same multigene family (**Figure 1C**). Other genes from the cluster represent CYP450, endonuclease, Na/H exchanger, phosphatase, two Small Secreted Proteins (SSP), and proteins with unknown function (**Supplementary Table S4**). This genomic organization of subtilases was not detected in other oomycetes genomes when using FungiDB or OGOB synteny searches (Basenko et al., 2018; McGowan et al., 2019), despite the presence of orthologous genes both in Saprolegniales and Peronosporales orders. The absence of similar gene clusters in the animal pathogenic species from the *Aphanomyces* genus supports the hypothesis that the duplication of secreted proteases happened during adaptation to the host plant. Taken together, these data suggest that within *A. euteiches*, the secreted proteases are pathogenicity factors that evolved through tandem duplication events. This evolution could offer greater flexibility for a broad-range pathogen such as *A. euteiches.*

### *A. euteiches* secreted proteases are induced during the infection process

**Figure 2:**
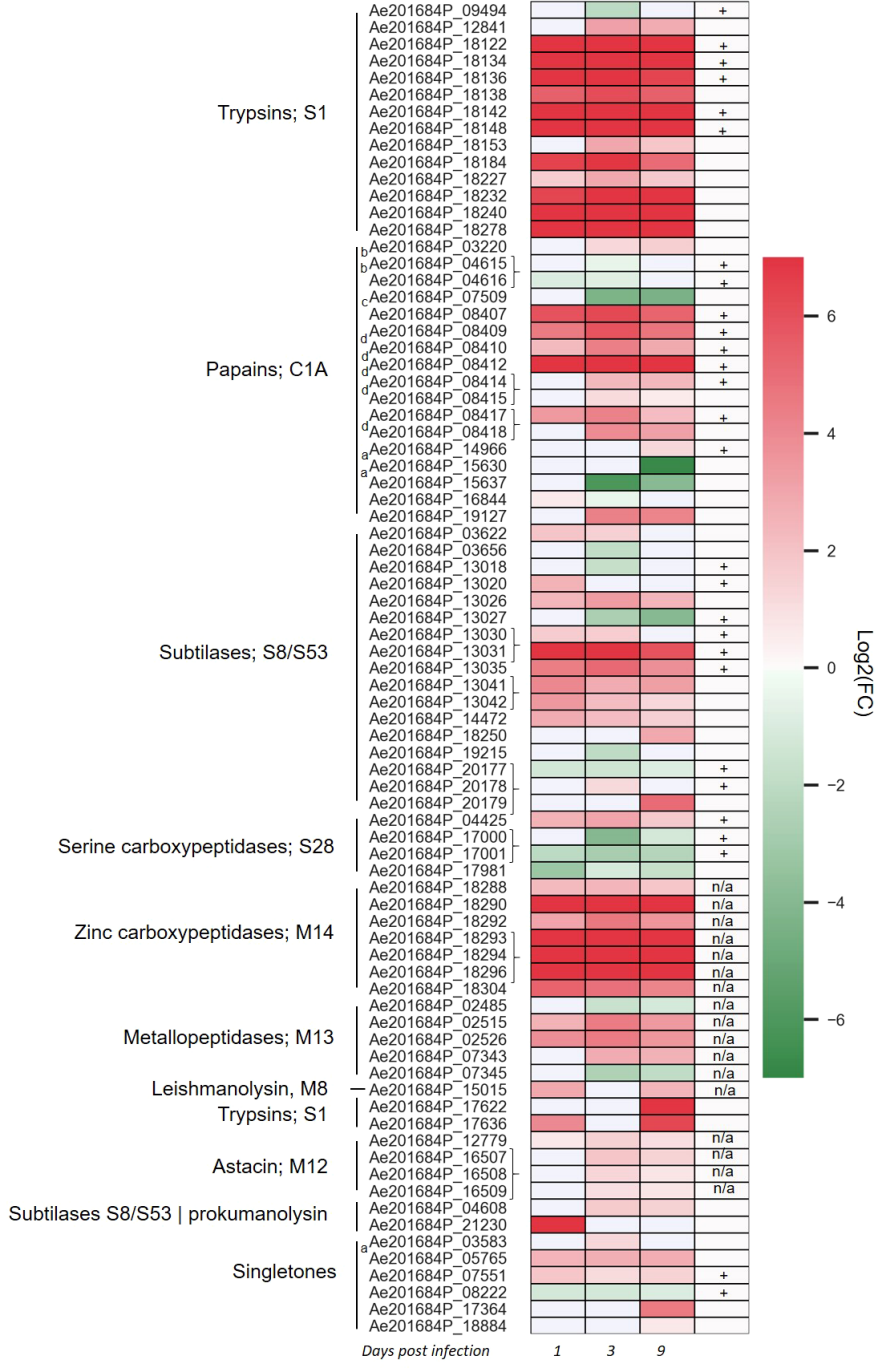
*Aphanomyces euteiches* differentially expressed genes coding for secreted proteases during *Medicago truncatula* infection. Three first columns of the heatmap represents log2(Fold Change) value of the significantly differentially expressed genes (DEG, p-value <0.05) during the time course of infection of *M. truncatula* roots (1, 3, 9 days post infection) as compared to the free-living mycelium. Braces on the right side of the gene name indicate adjacent tandemly repeated genes. The fourth column represents the identification of secreted proteases in activity-based proteome profiling proteomics (ABPP-MS) experiment using probes against active serine and cysteine hydrolases (+: identified by proteomics, blank: not identified by proteomics, n/a: probe not adapted for proteomics detection). Letter on the left side of the gene name indicate the presence of a binding domain within the secreted proteases [a: PAN/Apple domain (PF14295), b: ML domain (PF02221), c: fungal cellulose binding domain (PF00734), d: cysteine rich secretory domain CAP (PF00188)].

To identify whether there is a transcriptional regulation of secreted proteases during infection of the host plant, dual RNA-Seq of the infection process of *A. euteiches* on susceptible *Medicago truncatula* line was analyzed (Gaulin et al., 2018). Overall, 118 secreted proteases were expressed, among which 79 were differentially expressed (DE, adjusted p-value <0,05) at 1-, 3- or 9-days post infection (dpi) as compared to a mycelium grown on synthetic media (**Figure 2** and **Supplementary Table S5**). Several expression pattern can be distinguished. One includes almost all the trypsin S1 and zinc carboxypeptidase M14 genes, which are induced from 1 to 9 dpi. A second pattern identified around half of subtilase S8/S53, metallopeptidase M13 and of papain-like cysteine protease (PLCPs) belonging to protease family C1A, which are differentially upregulated overtime, while few genes are downregulated. Finally, some genes as subtilase S8 or trypsin S1 are respectively only express at the early stage of the infection or at a later stage. The tandemly repeated proteases do not show a common expression pattern since most of the astacin, M12 are slightly express from 3 to 9 dpi as the tandemly repeated C1A PLCPs, when the repeated subtilase S8 or zinc carboxypeptidase M14 genes are highly express at all stages. The transcriptomics evidence of massively upregulated secreted proteases during infection of *M. truncatula* roots underpins the role of these genes as pathogenicity factors in *A. euteiches.* In addition, the various expression pattern observed within similar proteins of the same multigene family suggests an independent transcriptional regulation and function of the tandemly repeated secreted proteases.

### *A. euteiches* secretes active serine hydrolase and cysteine proteases into plant apoplast during pea infection

**Figure 3:**
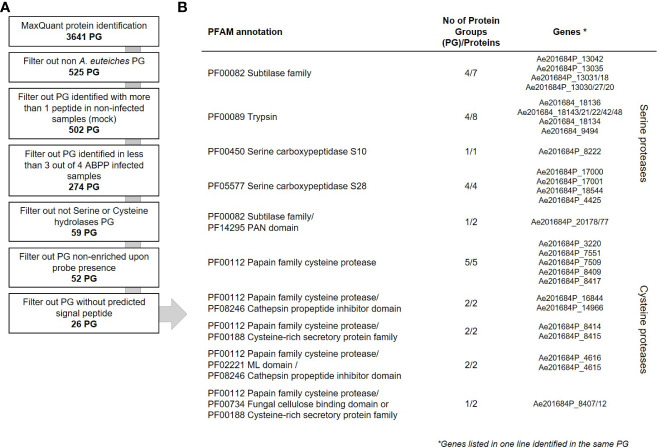
Data analysis workflow of secreted proteases from *A. euteiches.* **(A)** Downstream analysis of the MaxQuant assigned Protein Groups (PG). The number of PGs on each step is indicated in bold. **(B)** List of the mass spectrometry identified extracellular serine and cysteine proteases from *A. euteiches* present in apoplastic fluid of pea, 15 days post infection. Gene IDs according to AphanoDB nomenclature. Note that the sequences given in each line of the table belong to the same PG. See **Supplementary Tables S6**–**S8** for complete data.

To evaluate the contribution of *A. euteiches* extracellular proteases during legume infection, we hypothesized that secreted proteases should be present within the apoplast of infected roots. A semi-sterile pathosystem using *Pisum sativum* was established to collect sufficient volume of apoplastic fluid (AF), after roots infection by *A. euteiches* (**Supplemental Figure 1**). To perform the ABPP-MS assay, isolated apoplastic fluids (AF) were incubated with a cocktail of commercially available FP-biotin and DCG-04 to label Ser hydrolases and PLCPs, respectively. To further identify natively biotinylated proteins, samples without probes were generated (NPC = No-Probe-Control) and all were subjected for mass spectrometry. Protein identification was performed with MaxQuant software using the latest genome assembly of *A. euteiches* (Kiselev et al., 2022) and *P. sativum* (Kreplak et al., 2019). The analysis revealed a total of 3641 proteins groups (PG) (**Figure 3A**). PGs can represent several similar proteins, which are not distinguishable from detected peptides and tandemly repeated *A. euteiches* proteins. The PGs containing *P. sativum* proteins were filtered out (525 PG), and 20 PGs having similarity with *A. euteiches* within non-infected samples (mock) were removed. For further analysis, 274 PGs were kept, which were detected in at least three out of four replicates of the infected samples. From the resulting list, 59 PGs were carrying a serine or cysteine hydrolase domain (**Supplementary Table S6**), and 52 of these were enriched in the probe samples when compared to the no-probe-control (p-value <0.05). Among the 52 PGs, 26 were predicted as secreted leading to a final set of 35 proteins (**Supplementary Table S7**). A large majority of the corresponding genes are differentially expressed (28) at least at one time point during the infection of *M. truncatula* roots (**Figure 2**). Overall, from the 115 annotated Cys and Ser hydrolases that could be labelled with the probes, 99 (~85%) have a transcript in at least one of the time points of the infection. Therefore, the ABPP-MS approach allows identification of 30% of the *A. euteiches* expressed sequence during legume root colonization.

The set of the MS-identified secreted proteins consists of 4 PGs with subtilases that include seven proteins; four PGs with trypsins (eight proteins), one PG with Ser carboxypeptidase S10 (one protein); four PGs with Ser carboxypeptidase S28 (four proteins); five PGs with PLCPs (five proteins) (**Figure 3B**). In addition, six PGs include eight secreted modular proteins, in which an additional PFAM domain is associated with the proteolytic domain at the C-terminus. These correspond to: one PG with a subtilase connected to a PAN domain (PF14295, two proteins); two PGs with a PLCP connected to a Cys-rich secretory protein-CAP (PF00188, two proteins); two PGs with a PLCP connected to a ML domain (PF02221, two proteins) and one PG of a PLCP connected to a fungal cellulose binding domain (PF00734, one protein) or to a ML domain (PF02221, one protein). All the MS-identified serine subtilases are present in one gene cluster located in contig 762 presented in **Figure 1C**. The trypsin proteases originated also from one gene cluster in contig 60. A third cluster present on contig 595, corresponds to the PLCPs with or without an additional PFAM domain. Furthermore, two couples of tandemly repeated proteins were identified: carboxypeptidases (Ae201684P_17000 and _17001) and PLCPs-CAP proteins (Ae201684P_8414 and _8415). Both proteins of each of repeats are identified as a separate PG indicating their presence in the sample. Taken together, the ABPP-MS approach supports the prediction of proteases gene clusters and tandemly repeated sequences in *A. euteiches* genome.

### *A. euteiches* produces modular extracellular serine and cysteine proteases during legume infection

**Figure 4:**
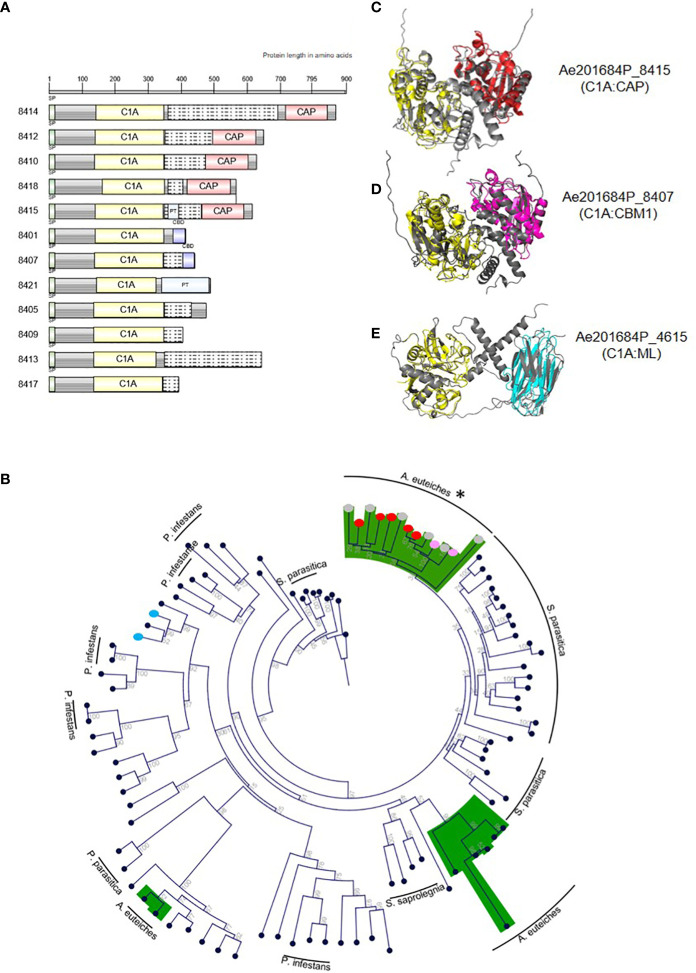
Modular papain-like cysteine proteases (PLCP) from *A. euteiches* identified in pea apoplastic fluids. **(A)** Protein domain architecture of the clustered PCLPs from *A. euteiches.* C1A: cysteine protease domain type C1A (PF00112); CBD: carbohydrate-binding module CBM1 (PF00734), CAP: Cysteine-rich secretory protein (PF00188), PT: PT-repeat (PF04886). Grey boxes: no domain predicted, Grey boxes with dashed lines: disorder region predicted by InterProScan. Scale = amino acids. **(B)** Phylogenetic tree constructed using the predicted C1A domain (PF00112) present in PLCPs from the plant pathogen *Phytophthora infestans*, the animal pathogen *Saprolegnia parasitica* and *A. euteiches*. Green color indicates the main group for *A. euteiches.* Asterik identified the clustered extracellular PCLPs of the pathogen. Colored dots indicated the associated domain of multidomain PLCPs of *A. euteiches* (red: CAP; pink: CBD; blue: ML-domain; grey: no domain). Neighbor-Joining method was used and boostrap are indicated **(C-E)**. PyMol representation of selected *A. euteiches* modular C1A cysteine proteases (grey). 3D structures superposition with a reference domain (colored) was performed, **(C)** Ae201684P_8415, cysteine protease C1A (PDB 1BP4, yellow), CAP domain (PDB 1SMB, red). RMSD scores: C1A domains = 0.877, CAP-domains = 1.077; **(D)** Ae201684P_8407, cysteine protease C1A (PDB 1BP4, yellow), CBM1 domain (PDB 5X34, magenta). RMSD scores: C1A domains 0.787, CBM1-domains = 0.937; **(E)** Ae201684P_4615, cysteine protease C1A (PDB 1BP4, yellow), ML-domain (PDB 1AHM, cyan). RMSD scores: C1A domains 0.674, ML-domains = 4.475. Structural predictions were performed using AlphaFold2.

Most families of fungal, oomycetes serine or cysteine proteases correspond to a single-domain protein (Muszewska et al., 2017). The identification by MS of multidomain extracellular proteases may suggest a specific function for these enzymes for *A. euteiches* invasion. The identified multidomain proteases harbor an additional binding region consisting of a PAN/Apple domain (PF14295) for subtilases and a ML lipid binding domain (PF02221), a Cys-rich secretory CAP domain (PF00188) or a CBM1 fungal cellulose binding domain (PF00734) for PLCPs. PAN/Apple and CBM1 have been suggested to mediate protein/carbohydrate or protein/protein interactions (Tordai et al., 1999), while CAP and ML domains are related to sterol and lipid binding capacities, respectively (Inohara and Nuñez, 2002; Schneiter and Di Pietro, 2013). InterProScan domain architecture searches revealed the large distribution within eukaryotes of modular PAN-trypsin proteases with a large representation in animals, but the association of a PAN/Apple domain with a subtilase is unique to the *Aphanomyces* genus. The combination of a cysteine C1A domain (PF00112) with a lipid-binding ML domain (C1A:ML) is present in several Stramenopila, including oomycetes, the yellow-green algae (Xanthophyceae, *Tribonema minus*), and brown algae (Phaeophyceae, *Ectocarpus siliculosus*), but the C1A:CBM1 and C1A:CAP associations are restricted to the *Aphanomyces* genus. Only heterotrophic Amoebozoan slime molds protists (*Planoprotselium fungivorum, Dictyostelium purpureum, Polysphondylium pallidum*) harbor predicted extracellular proteins with domains organized in the reverse order (e.g. CAP:C1A). The gene cluster encoding PLCPs identified by MS displays the unique structure of modular cysteine proteases. Within 50 kb on contig 595, this gene cluster contains 12 extracellular PLCP-encoding genes, which have a conserved C1A domain at the N-terminal region associated with a variable C-terminal region consisting either of CBM1, CAP or no domains. The domains are commonly separated by a disordered linker often represented as a PT-repeat (**Figure 4A**). Phylogenetic analysis of C1A-domain from PCLPs sequences from *P. infestans*, *S. parasitica* with *A. euteiches*, identified 12 multidomain members of the clustered-PLCP of the root pathogen in one group derived from a unique C1A-containing protein (**Figure 4B**). Within this group, CAP and CBM1 additional domains form two subgroups, suggesting a first duplication of the catalytic domain followed by acquisition of an additional ‘binding’ module for modifying the initial function of the C1A domain. (**Figure 4B**). The others C1A-containing proteins of *A. euteiches* are mainly detected in two groups, related to the fish pathogen *S. parasitica* with the exception of C1A-ML multidomain PLCPs more related to *P. infestans.* To predict whether the additional domain within the original PLCPs may modulate the activity of the corresponding enzyme, the structure of the catalytic and binding domains of each protease was predicted with Alpha-Fold2 (Jumper et al., 2021). Superpositions of the predicted 3D modelling with a reference structure for each domain are shown in **Figures 4C–E**. All the modular proteins keep a structural homology (RMSDE score <= 1) with the reference structure. Despite a slightly higher RMSDE score of ~4, the structural alignment of the ML-domain also revealed a structural topology to immunoglobulin (PDB 1AHM). According to the modelling results, the additional binding domain detected in the extracellular PCLP of *A. euteiches* may serve for the adhesion of a protease to a specific substrate to enhance its activity during infection.

## Discussion

The genome of the detrimental-roots colonizing filamentous oomycete *A. euteiches* is predicted to have a large set of proteolytic enzymes (Kiselev et al., 2022). Here we explored the genomic organization of protease sequences, their expression during host infection, and characterized whether they are present and active during root colonization using an ABPP-MS approach. We identified original modular secreted serine subtilisin and PCLPs in the apoplast of infected roots that may contribute to *A. euteiches* pathogenicity.

The curated annotation of the predicted proteases from the long-read sequenced ATCC201684 strain of *A. euteiches* showed that the proteases consist mainly of trypsin (serine protease, S1 class) and papain (cysteine protease, C1 class) families. Up to 60% of secreted protease genes were found tandemly repeated and frequently organized in large clusters enriched in proteases (e.g. over 50% of genes within a cluster encode proteases). McGowan et al. (2019) identified that 40% of the 20 oomycete species analyzed, displayed GO terms enrichment in terms linked to pathogenicity such as ‘catalytic activity, acting on protein’ (GO:140096) and ‘peptidase activity’ (GO:0008233), in tandem duplicate genes. Tandem gene duplication in combination with homologous recombination are postulated to accelerate pathogenicity factors evolution within oomycete genomes (Haas et al., 2009; Fitzpatrick et al., 2010; Liang et al., 2020). In *A. euteiches*, we suspected that neo-functionalization occurs after tandem duplication of the secreted cysteine protease family, due to the presence at the C-terminal part of the enzymes of various additional domain associated either to carbohydrate-binding capacity (CBM1, PAN/Apple) or to sterol/lipid affinity (ML, CAP).

The whole pathogen’s transcriptome analysis of *M. truncatula* roots infected by *A. euteiches*, revealed induction of serine (trypsin, subtilisin), PLCP and zinc carboxypeptidases (M14) during infection. Most of the serine proteases and PLCP showed induced expression from the first day of infection, with an increase in the number of induced genes in 3 and 9 dpi, suggesting their key role in plant invasion. The differential pattern of expression is likely related to the hemibiotrophic life style of the pathogen. From one day to six day after infection the pathogen colonized almost all the cortex root tissues of *M. truncatula*, before invading the stele and vascular tissues in fifteen days, causing root rot symptoms (Djébali et al., 2009). Thus, before turning necrotrophic, extracellular peptidases of *A. euteiches* can contribute to the degradation of host proteins located into the apoplast or structural proteins from the plant cell wall. At later stage of the infection, secreted proteases may counteract apoplastic immunity through degrading host-derived defense proteins, be directly toxic for the root tissues or have a role for nutrient acquisition by digesting host tissues.

Several studies on plant-microbe interactions (van der Hoorn et al., 2004; Meijer et al., 2014) have reported the presence of plant proteases within infected tissues, and only few microbial proteases have been functionally characterized. The developed ABPP-MS assay on apoplastic fluid from pea roots infected by *A. euteiches* using probes that target serine (FP) and cysteine (DCG04) proteases, allows the identification of 35 *A. euteiches* extracellular active proteases. This set of active enzymes covers ~30% of total number of expressed genes during *M. truncatula* infection, demonstrating the efficiency of ABBP-MS assay to identify putative pathogenicity factors. The remarkable signature of the identified proteases in the apoplastic fluid of infected-pea roots, correspond to multidomain proteases with an additional ‘binding domain’ having affinity for carbohydrates or lipids/sterols. Eukaryotic proteases are rarely associated with a non-catalytic domain, but *A. euteiches* produces several different combinations of extracellular multidomain proteases: serine proteases with PAN/Apple domain and cysteine proteases with CBM1, ML, CAP domains. Some domain combinations, like C1A:CBM1 and C1A:CAP, are only detected in the genus *Aphanomyces*. In addition, twelve of C1A-multidomain proteases identified by MS are organized in one cluster within the genome of *A. euteiches*. The members of the cluster are found in one phylogenetic group divided in two classes with single or multidomain PCLPs, suggesting independent acquisition of the additional ‘binding’ domain. The other PLCPs of *A. euteiches* are identified in two main groups related to the fish pathogenic oomycete *S. parasitica*, except the C1A:ML multidomain proteases which are more closely related to single domain proteases of the plant pathogen *P. infestans.* Structural prediction of the modular C1A proteases of *A. euteiches* indicates that the additional domain does not form a lid structure or an occluding loop that can cover the active site, suggesting the evolution of specialized functions for these PLCPs. Inappropriate activity of proteases can be deleterious to the cell or the organism that produces them, thus, proteases activity is regulated to allow proteolysis event only in an adapted environment or cellular compartment (for review see Kopitar-Jerala, 2012). Here we suggest that the non-catalytic protease-associated domain found in *A. euteiches* corresponds to regions responsible for regulation or targeting of the enzymes.

To conclude, the ABPP-MS approach allows the characterization of original active extracellular multidomain apoplastic proteases from a soil-borne oomycete that could play a key role in root infection. This system can be easily translated to other pathosystems and will facilitate addressing the global challenge in the selection of microbial candidate genes for functional analysis.

## Data availability statement

The datasets presented in this study can be found in online repositories. The names of the repository/repositories and accession number(s) can be found in the article/**Supplementary Material**.

## Author contributions

AK, EG, RH planned and designed the experiments. AK, LC, LOC performed the experiments. FK, MK performed proteomics analysis. AK, EG analysed the data. AK, EG wrote the manuscript with help of all authors. All authors contributed to the article and approved the submitted version.

## Supplementary material

Supplementary Figure 1 Semi-sterile *in vitro* system for ABPP-MS assay between *Pisum sativum cv Précovil* and *A. euteiches.* Roots of infected (left) and non-infected pea (right) at 15 days post infection with 10^5^ zoospores of *A. euteiches*. Plants were maintained at 21°C in a semi-sterile condition in pots filled with zeolite as a solid substrate and Fåhraeus media as the nutritive solution under 18h/6h-light/dark alternance. The black arrow points to root rot symptoms; scale bar = 1 cm. The cross sections of primary roots were stained with Wheat Germ Agglutinin-Alex Fluor 555 conjugate to detect *A. euteiches* hyphae (green). UV fluorescence reveals phenolic compounds (blue) and pericycle cells reinforcement (red arrow) in infected roots. Note that the pathogen is restricted to the root cortex as previously reported upon infection of a tolerant line of the model legume *Medicago truncatula* (i.e., Jemalong A17) by the same strain of *A. euteiches* (Djébali et al., 2009). Scale bar = 100 µm.
