# Supplementary figures and images for: The root pathogen Aphanomyces euteiches secretes modular proteases in pea apoplast during host infection
Source: Front Plant Sci. 2023 Mar 27;14:1140101. doi: 10.3389/fpls.2023.1140101 (PMC10084794; doi:10.3389/fpls.2023.1140101)

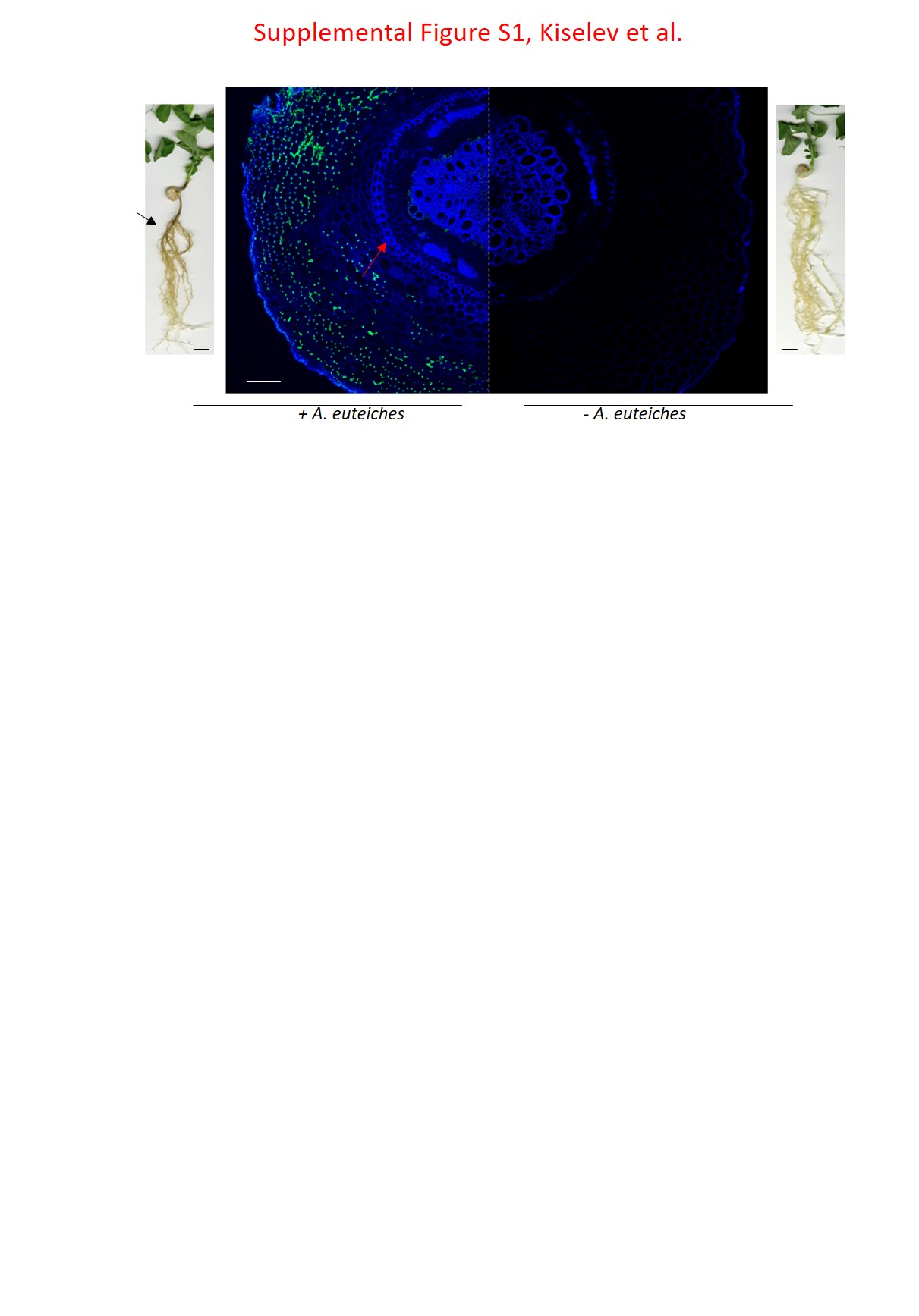

Supplement: Supplementary Figure 1 — Semi-sterile in vitro system for ABPP-MS assay between Pisum sativum cv Précovil and A. euteiches. Roots of infected (left) and non-infected pea (right) at 15 days post infection with 105 zoospores of A. euteiches. Plants were maintained at 21°C in a semi-sterile condition in pots filled with zeolite as a solid substrate and Fåhraeus media as the nutritive solution under 18h/6h-light/dark alternance. The black arrow points to root rot symptoms; scale bar = 1 cm. The cross sections of primary roots were stained with Wheat Germ Agglutinin-Alex Fluor 555 conjugate to detect A. euteiches hyphae (green). UV fluorescence reveals phenolic compounds (blue) and pericycle cells reinforcement (red arrow) in infected roots. Note that the pathogen is restricted to the root cortex as previously reported upon infection of a tolerant line of the model legume Medicago truncatula (i.e., Jemalong A17) by the same strain of A. euteiches (Djébali et al., 2009). Scale bar = 100 µm. [file Image_1.jpeg]
